# Supplementary material for: Impact of Coenzyme Q10 Supplementation on Skeletal Muscle Respiration, Antioxidants, and the Muscle Proteome in Thoroughbred Horses
Source: Antioxidants (Basel). 2023 Jan 24;12(2):263. doi: 10.3390/antiox12020263 (PMC9951987; doi:10.3390/antiox12020263)
Supplement: Supplementary file 1 [file antioxidants-12-00263-s001.zip › antioxidants-2106799-supplementary.pdf]

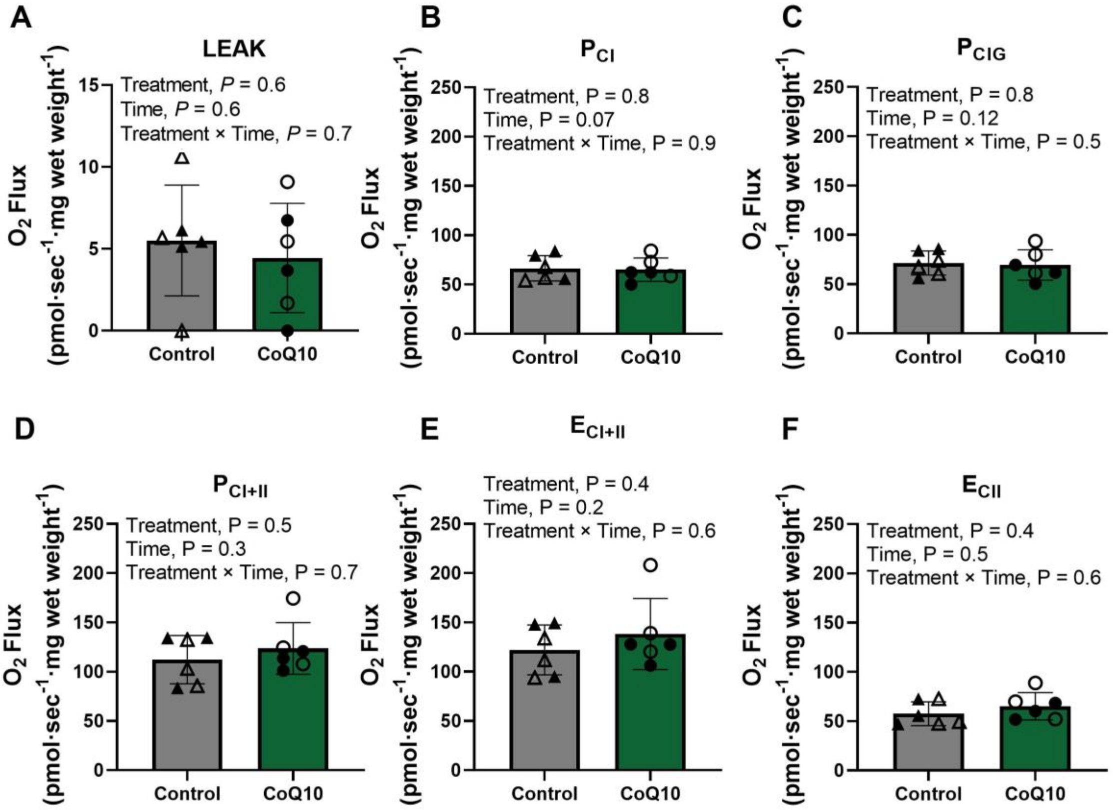

Figure S1. Integrative (per mg tissue) mitochondrial capacities in the gluteus medius of fit Thoroughbred horses before and after 30 d supplementation of CoQ10 or control diet. Open circles represent horses on the CoQ10 supplement during the first supplementation period and closed circles represent horses on the CoQ10 supplement during the second supplementation period. Open triangles represent horses on the control diet during the second supplementation period and closed triangles represent horses on the control diet during the first supplementation period.
